# Socio-Economic Factors of Bacillary Dysentery Based on Spatial Correlation Analysis in Guangxi Province, China

**DOI:** 10.1371/journal.pone.0102020

**Published:** 2014-07-18

**Authors:** Chengjing Nie, Hairong Li, Linsheng Yang, Gemei Zhong, Lan Zhang

**Affiliations:** 1 School of Public Administration and Policy, Hebei University of Economics and Business, Shijiazhuang, China; 2 Institute of Geographic Sciences and Natural Resources Research, Chinese Academy of Science, Beijing, China; 3 Institute of Environmental Health and Endemic Disease Prevention, Guangxi Center for Disease Prevention and Control, Nanning, Guangxi; 4 Institute for Environmental Health and Related Product Safety, Chinese Center for Disease Control and Prevention, Beijing, China; State Key Laboratory of Pathogen and Biosecurity, Beijing Institute of Microbiology and Epidemiology, China

## Abstract

**Background:**

In the past decade, bacillary dysentery was still a big public health problem in China, especially in Guangxi Province, where thousands of severe diarrhea cases occur every year.

**Methods:**

Reported bacillary dysentery cases in Guangxi Province were obtained from local Centers for Diseases Prevention and Control. The 14 socio-economic indexes were selected as potential explanatory variables for the study. The spatial correlation analysis was used to explore the associations between the selected factors and bacillary dysentery incidence at county level, which was based on the software of ArcGIS10.2 and GeoDA 0.9.5i.

**Results:**

The proportion of primary industry, the proportion of younger than 5-year-old children in total population, the number of hospitals per thousand persons and the rates of bacillary dysentery incidence show statistically significant positive correlation. But the proportion of secondary industry, per capital GDP, per capital government revenue, rural population proportion, popularization rate of tap water in rural area, access rate to the sanitation toilets in rural, number of beds in hospitals per thousand persons, medical and technical personnel per thousand persons and the rate of bacillary dysentery incidence show statistically significant negative correlation. The socio-economic factors can be divided into four aspects, including economic development, health development, medical development and human own condition. The four aspects were not isolated from each other, but interacted with each other.

## Introduction

Bacillary dysentery, caused by Shigella bacteria, is a bacterial infection of intestines which may result in severe diarrhea [1]–[3]. The infection is spread from person to person via oral-feces, food or drinking water. Epidemics are frequently occurred in overcrowded populations with poor sanitation and most cases occur in summer and autumn [1], [4]. Bacillary dysentery is still a public health problem in China, especially in Guangxi Province. The incidence of bacillary dysentery has been reported to exhibit distinct regional differences in China, with the highest incidence of 132.37 per 100,000 in Beijing, and lowest incidence being reported in Fujian and Shanghai Provinces in 2011. In different regions, the main influence factors of bacterial dysentery were different [5], [6]. In this paper, the socio-economic factors of bacillary dysentery were analyzed based on regional differences in Guangxi Province. Though the morbidity and the mortality of bacillary dysentery have decreased considerably in Guangxi since the 1990s, a considerable burden still exists, particularly among the children and the older people with low economic status [7], [8]. Chinese Government has developed a strategic plan for the national surveillance of bacillary dysentery [9], [10], but the risk of bacterial dysentery has increased in recent years, and it has attracted wide attention of scholars in China and abroad [9].

In the past decade, the relationship between the bacillary dysentery and the meteorological factors has been reported in many studies [4], [11]–[16], The seasonality of bacillary dysentery incidence indicated that meteorological factors might play an important role in its epidemiology [12], [17]. But no significant progress has been made in terms of the model investigating the socio-economic factors of bacillary dysentery. In Guangxi Province, the socio-economic factors, such as public health and the level of per capita income, played an important role in bacillary dysentery's epidemiology, control and prevention [18].

For time series data, multiple regression model is a good choice [15]. While for regional differences, the spatial correlation analysis has its unique advantage [19], [20]. Spatial correlation have been widely used in health studies [21]–[23], but there is few study about spatial variations of social-economic impacts on bacillary dysentery in Guangxi. In this paper, global spatial autocorrelation was employed, which indicated whether bacillary dysentery and socio-economic factors were randomly located over the study area, or followed some spatial pattern, thereby indicating some underlying process [24]. Through the spatial correlation analysis on bacillary dysentery and its socio-economic factors in Guangxi Province, the main risk factors of bacillary dysentery can be found, and the corresponding control measures were also put forward in this paper.

## Data and Methodology

### Data sources

**Figure 1:**
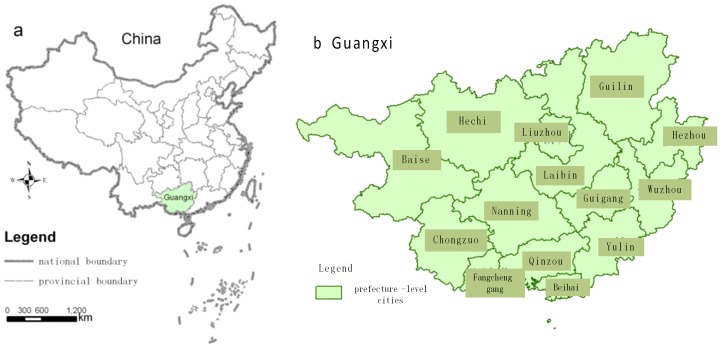
Administrative map of Guangxi Province.

The epidemiological case data, population data and socioeconomic data were obtained from Guangxi Province, China. Guangxi Province is in the Pearl River basin of southern China (Figure 1a, 1b), from 20° 54′ to 26° 23′ N and 104° 29′ to 112° 03′ E. Guangxi has a subtropical climate, and usually has heavy rain in the summer. The summers are generally long and hot. Average annual temperature is 17–23°C, and average annual precipitation is 1250–1750 mm. Guangxi has extremely complex geological features, with significant differences between regions.

Bacillary dysentery is a legally mandated notifiable disease in China. All clinical and hospital doctors are required to report cases of bacillary dysentery to local Centers for Diseases Prevention and Control. In this study, both clinical and laboratory diagnosed cases were collected. Bacillary dysentery is a very common disease in China, then it is not difficult for doctors to make a correct clinical diagnosis. Therefore, in different regions of Guangxi Province, it is believed that the disease data quality is reliable. Monthly-notified cases of bacillary dysentery were provided by Guangxi Center for Diseases Prevention and Control. This retrospective study was approved by Guangxi Center for Disease Prevention and Control. The written informed consent was not obtained. The patient records information has been anonymized and de-identified prior to analysis. The study period covered 2 years (2009–2010).

Demographic data for Guangxi were collected from local government reports. The socio-economic data were collected from Guangxi Statistical Yearbook, Guangxi Health Statistics Yearbook and Guangxi Civil Affairs' Statistical Yearbook. The data were analyzed at a county-level.

### Methodology

The spatial autocorrelation concept is based on the first geography law introduced by Tobler [25]. “Everything is related to everything else, but nearest things is more related than distant things”. Global indicators of spatial autocorrelation measure if and how much the dataset is autocorrelated throughout the study region. In the field of infectious diseases, spatial autocorrelation measures the degree of dependency among the incidence of infectious disease in different areas, considering their similarities and their distance relationships at the same time.

In this paper, the relationship between bacillary dysentery and related socio-economic factors were measured based on spatial autocorrelation at county level in Guangxi Province. In general, the first step is to establish the corresponding weight matrix; followed by visual analysis, to obtain the basic distribution of the attribute; the last step is the global correlation analysis, including single variable space autocorrelation analysis and multivariate spatial autocorrelation analysis. In this paper, only the multivariate spatial autocorrelation analysis was implemented, since the research objective was to explore the main socio-economic factors of bacillary dysentery. The spatial multivariate autocorrelation was based on the software of ArcGIS10.2 and GeoDA 0.9.5i.

The variables of socio-economic driving forces were proportion of primary industry, proportion of secondary industry, proportion of tertiary industry, per capital GDP, per capital government revenue, sex ratio of male and female, percentage of illiterate population in total population aged 15 and above, rural population proportion, rate of younger than 5 year old children in total population, popularization rate of tap water in rural area, access rate to the sanitation toilets in rural, number of hospitals per thousand persons, number of beds in hospitals per thousand persons, medical and technical personnel per thousand persons. One of the principal global indicators of autocorrelation is the Moran's index I [26], defined in formula as follows,
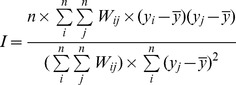



Where 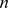 is the total pixel number, 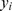 and 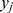 are intensities in points 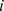 and 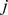 (with 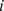
≠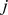) respectively, 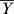 is the average value, and 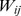 is an element of the weight matrix.


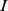
∈ [−1; 1]; if 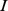
∈ [−1; 0] there is a negative autocorrelation; if 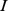
∈ [0; 1] there is a positive autocorrelation. Theoretically, if 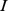 converges to 0, there is null autocorrelation, in most of the cases, instead of 0 the value used to affirm the presence of null autocorrelation is given in equation:
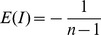
where 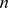 is the number of events in the whole distribution

The credibility test of Moran's I is calculated as
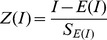


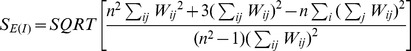



Moran's I statistics have been widely used in social and health studies [21]–[23]. A few applications have been presented from epidemic in recent years [27]–[29]. Most epidemic phenomena displayed geographic patchiness, and it was found at all spatial scales – from micrometers to continental and ocean-wide scales showed that the Moran's I of epidemic incidence data was scale-dependent [30], [31]. It was found that the occurrence of spatial autocorrelation of epidemic was highly dependent on the aggregation level of the natural and socio-economic data. This paper presented a study of Moran's I on bacillary dysentery as one of the few early attempts. Moreover, this paper used Moran's I statistics to explain some remaining questions from previous publications about bacillary dysentery, and the method of Monte Carlo simulation was used to test the confidence level of Moran's I.

## Results and Discussion

### Spatial distributions of socio-economic factors

**Figure 2:**
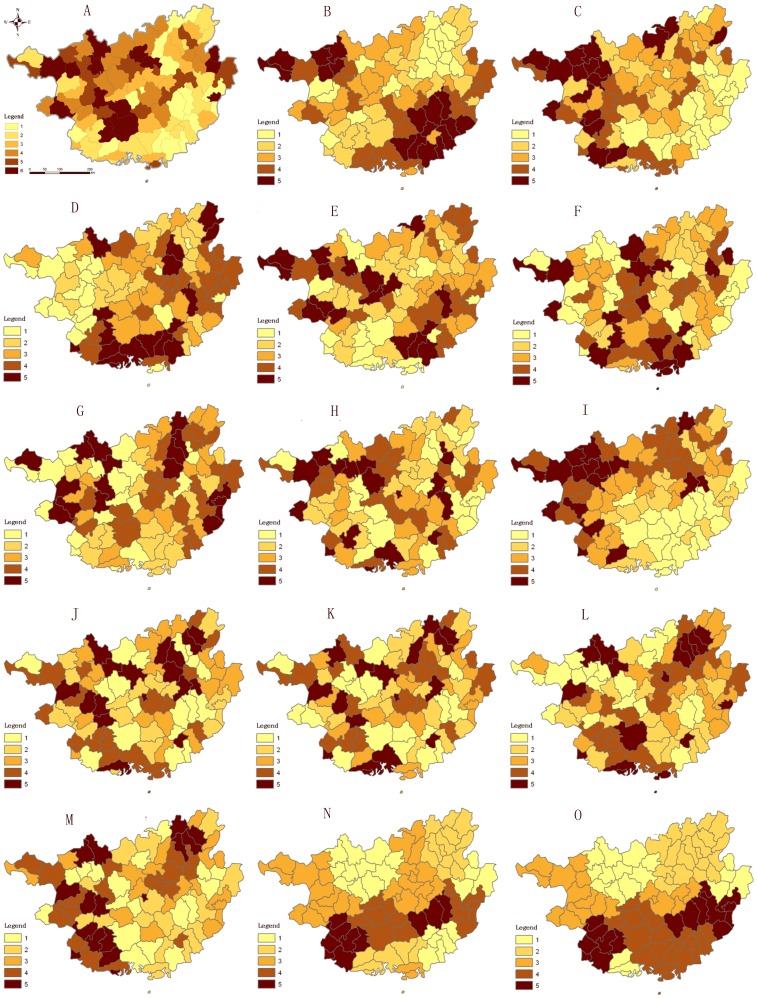
Spatial distributions of bacillary dysentery incidence and socio-economic factors.

Prior to the analysis of global spatial autocorrelation, it was necessary to understand the regional distribution of each variable (Figure 2). The areas with high incidence of bacillary dysentery were distributed in the northwest of Guangxi, Nanning city and its surrounding districts, Hezhou city and its surrounding districts (Figure 2A). The 14 socio-economic attributes were distributed as follows: The areas with high percentage of Children younger than 5 year old in total population were mainly located in the southeast and northwest parts of Guangxi (Figure 2B); those with high percentage of illiterate population in total population aged 15 and above in the northwest, and parts of the southeast and northeast of Guangxi (Figure 2C); and those with high sex ratio of male and female in the south and east of Guangxi (Figure 2D); the rural population more dispersedly with no significant aggregation (Figure 2E); the areas with high percentage of primary industry in the south and part of the north in Guangxi (Figure 2F); the areas with high percentage of secondary industry in the east and parts of northwest (Figure 2G); and the areas with high percentage of tertiary industry in the northwest (Figure 2H).

The areas with large number of hospitals per thousand persons were located in parts of northern Guangxi (Figure 2I). The number of beds in hospitals per thousand persons, and medical and technical personnel per thousand persons are dispersed (Figure 2JK); the areas with high per capital GDP were mainly located in the southwest and northeast (Figure 2L); those with high per capital government revenue in parts of west and northeast of Guangxi (Figure 2M); and those with high popularization rate of tap water in rural area (Figure 2N) and access rate to the sanitation toilets in rural in the south of Guangxi (Figure 2O).

### Spatial correlation of bacillary dysentery and the socio-economic factors

The spatial correlation between bacillary dysentery and the socio-economic factors were executed (Table 1).

**Table 1:** Morian's I and the confidence level between the morbidity of bacillary dysentery and the socio-economic factors.

Index	Morian's I	Confidence Level(%)
Proportion of Primary Industry	0.069	94.2
Proportion of Secondary Industry	−0.055	90.8
Proportion of Tertiary Industry	0.005	0.1
Per Capital GDP	−0.041	78.3
Per Capital Government Revenue	−0.033	67.2
Sex Ratio of Male and Female	0.022	31.5
Percentage of Illiterate Population in Total Population Aged 15 and above	0.020	33.3
Rural Population Proportion	0.024	34.6
Rate of Children Younger than 5 Year Old in Total Population	0.044	73.6
Popularization Rate of Tap Water in Rural Area	−0.121	99.8
Access Rate to the Sanitation Toilets in Rural Area	−0.114	99.8
Number of Hospitals per Thousand Persons	0.038	68.7
Number of Beds in Hospitals per Thousand Persons	−0.030	64.3
Medical and Technical Personnel per Thousand Persons	−0.025	60.7

The proportion of primary industry and the rate of bacillary dysentery incidence showed a positive correlation at 94.2% confidence level, a higher degree of confidence. The proportion of secondary industry and the rate of bacillary dysentery incidence showed a negative correlation at 90.8% confidence level, a higher degree of confidence. The spatial correlation between the proportion of tertiary industry and bacillary dysentery incidence was not significant. The reasons might be that in Guangxi the agricultural machinery production level was not high, agricultural labors relied on input, while the low-level sanitary conditions of farmers' working environment caused susceptibility to bacillary dysentery and workers' working environment was better than farmers' [3], [18]. The tertiary industry included a variety of career types without susceptibility to bacillary dysentery.

Per capital GDP, per capital government revenue and the rates of bacillary dysentery incidence showed a negative correlation at about 70% confidence level, a general degree of confidence. Per capital GDP and per capital government revenue are representative of the level of economic development. Thus, the negative correlation means good economy is helpful for the improvement of health conditions and then reduction of the sensitivity to bacillary dysentery.

Sex ratio of male and female, percentage of illiterate population in total population aged 15 and above, rural population proportion and the rate of bacillary dysentery incidence showed a positive correlation at about 30% confidence level, a poor degree of confidence. Thus, the sex ratio of male and female, percentage of illiterate population in total population aged 15 and above, rural population proportion might increase the bacillary dysentery incidence. For these variables, the regional differences were not obvious, and this may be the reason why the confidence degree was poor.

Rate of children younger than 5 year old in total population and the rate of bacillary dysentery incidence showed a negative correlation at 73.6% confidence level, a general degree of confidence. Therefore, a higher percentage of children younger than 5 year old was related to a higher incidence of bacillary dysentery, which was because children had a low immunity and was more susceptible to infection of bacillary dysentery.

Popularization rate of tap water in rural area, access rate to the sanitation toilets in rural and the rate of bacillary dysentery incidence showed a negative correlation at 99.8% confidence level, a very high degree of confidence. The reason was that water pollution and poor sanitation were the main causes of outbreak and prevalence of bacillary dysentery [32], [33], so the improvement of drinking water and sanitary conditions will be contributed to decrease the incidence of bacillary dysentery.

Number of beds in hospitals per thousand persons, medical and technical personnel per thousand persons and the rate of bacillary dysentery incidence showed a negative correlation at over 60% confidence level, a general degree of confidence. This was because number of beds in hospitals per thousand persons, medical and technical personnel per thousand persons represented medical conditions, and better medical conditions were helpful for the control of spreading of the epidemic.

Number of hospitals per thousand persons and the rate of bacillary dysentery incidence showed a positive correlation at 68.7% confidence level, a general degree of confidence. The more hospitals per thousand persons were often accompanied with low-technology smaller hospitals, so the correlation was positive.

### Comprehensive analysis of the socio-economic factors of bacillary dysentery

**Figure 3:**
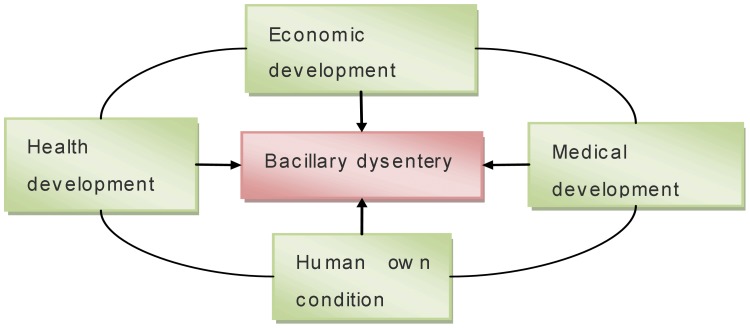
Socio-economic impact model of the occurrence and spread of bacillary dysentery.

In summary, the 14 socio-economic factors can affect the incidence of bacillary dysentery. The socio-economic factors can be divided into four aspects: economic development, health development, medical development and human own condition (Figure 3). Economic development includes proportion of primary industry, proportion of secondary industry, proportion of tertiary industry, per capital GDP and per capital government revenue; health development includes popularization rate of tap water and access rate to the sanitation toilets in rural areas; medical development includes number of hospitals per thousand persons, number of beds in hospitals per thousand persons, medical and technical personnel per thousand persons; and human own condition includes sex ratio of male and female, percentage of illiterate population in total aged 15 and above, rural population proportion, rate of children younger than 5 year old in total population. According to the former analysis, both health development and economic development play very important roles in the incidence of bacillary dysentery. Therefore, it is really necessary to increase the popularization rate of tap water in rural area, the access rate to the sanitation toilets in rural area and the level of economic development.

In addition, the four aspects of socio-economic factors are not isolated from each other, but interacted with each other. For example, the development of economy can improve the level of both health development and medical development. The development of health condition and medical condition can also improve the human environment which can attract more investment and then promote the development of economy. The economic development can enhance the education level, and reduce the proportion of agricultural population, and thereby reduce the incidence of bacillary dysentery. Therefore, it is necessary to improve all the four aspects. Only the progress of the four aspects can achieve more effects in prevention and control of bacterial dysentery.

## Conclusions

Through the spatial correlation analysis, the following conclusions can be drawn: Popularization rate of tap water in rural area, access rate to the sanitation toilets in rural and the rate of bacillary dysentery incidence showed a significantly negative correlation. The two factors were the most important risk factors for bacillary dysentery.

The proportion of primary industry and the rate of bacillary dysentery incidence showed a positive correlation. The proportion of secondary industry and the rate of bacillary dysentery incidence showed a negative correlation, which both factors played important roles in the epidemiology.

The socio-economic factors can be divided into four aspects: economic development, health development, medical development and human own condition. The four aspects are not isolated from each other, but interacted with each other. Therefore, it is necessary to improve all the aspects. Only the progress of the four aspects can achieve more effects in prevention and control of bacterial dysentery.
